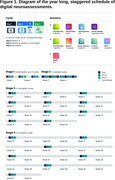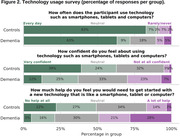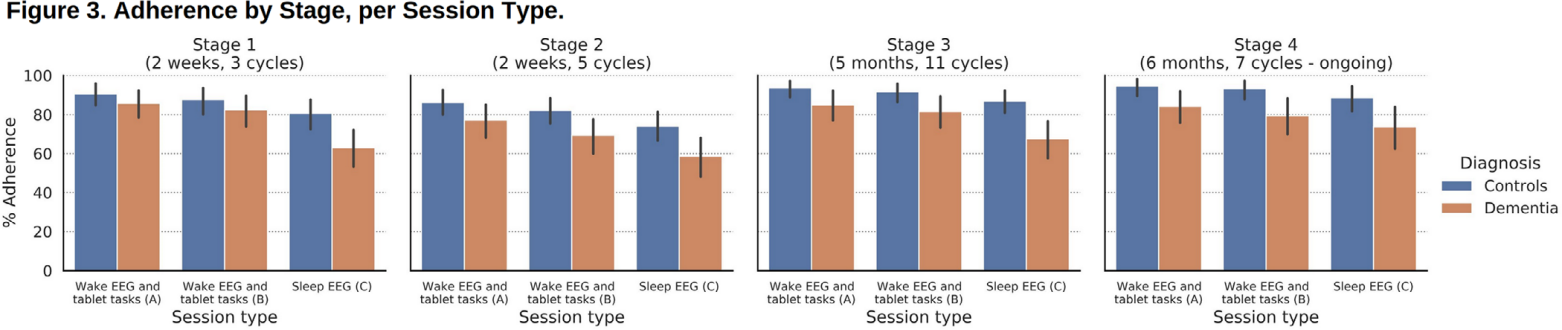# A longitudinal real‐world study in patients with Alzheimer’s Disease dementia using frequent multi‐domain digital measurements performed at‐home on the Cumulus Neuroassessment Platform: usability and feasibility findings

**DOI:** 10.1002/alz.094325

**Published:** 2025-01-09

**Authors:** Shannon Diggin, Azar Alexander‐Sefre, Brian Murphy, Hugh Nolan, James B Rowe, Laura M Rueda‐Delgado, Alison R Buick

**Affiliations:** ^1^ Cumulus Neuroscience, Belfast UK; ^2^ Cumulus Neuroscience, Dublin Ireland; ^3^ Department of Clinical Neurosciences, University of Cambridge, Cambridge UK

## Abstract

**Background:**

Many outcome measures used in AD clinical trials require clinic visits and are paper based, making them infrequent and burdensome ‘snapshots', subject to rater bias. A consortium of 10 pharma companies came together with Cumulus Neuroscience to design a solution for frequent, objective, real‐world measurement across a range of domains. We present a study that examined the feasibility of asking patients with mild dementia to use the neuroassessment platform repeatedly at home for one year.

**Method:**

Seven UK sites recruited Alzheimer’s type mild dementia patients (n = 59, ACE‐III scores >60 and = 88) and a matched cohort of controls (n = 60). Participants had 2 weeks of at‐home familiarisation to become comfortable with the 8 assessments presented on a mobile tablet and EEG headsets, one for wake and another for sleep. A staggered longitudinal protocol followed (Figure 1), with burst sampling tapering to periodic sampling over the year. Benchmark paper‐based assessments (including ADAS‐Cog) and self‐reported usability were collected at months 0, 6 and 12. Additionally, plasma was collected at months 6 and 12 for later biomarker analysis.

**Result:**

Preliminary results are available, with <1% expected at‐home sessions still outstanding (last patient out is scheduled for March 2024). Feedback collected at baseline (Figure 2) showed differences in technology usage. Patients felt they required more technology support than controls (49% vs 17%). Nonetheless, they were successful in using the study technology at home on a regular basis and maintained a high level of adherence to the protocol (Figure 3). Overall compliance was 72.9% for dementia, 87.5% for controls and was highest in the latter stages of the study during periodic sampling (Stage 4: 80.1% dementia, 92.6% controls). System Usability Scale results indicated controls rated usability higher (63.8) than dementia participants (54.5) at baseline, increasing for both groups by Week 26 (71.8 controls; 62.2 dementia).

**Conclusion:**

With appropriate technology design, and provision of training and support, patients with Alzheimer’s disease dementia are capable and willing to provide repeated, real‐world samples of a broad range of objective digital endpoints for clinical research.